# Effects of Cover Crops on Nematode Communities in Spinach Production

**DOI:** 10.3390/ijms252413366

**Published:** 2024-12-13

**Authors:** Elyse Aubry, Jerry Akanwari, Ping Liang, Walid Ellouze, Jonathan Gaiero, Tahera Sultana

**Affiliations:** 1 London Research and Development Centre, Agriculture and Agri-Food Canada, Vineland Station, ON L0R 2E0, Canada; 2 Department of Biological Sciences, Brock University, St. Catharines, ON L2S 3A1, Canada

**Keywords:** nematode, cover crop, Cowpea, Pearl Millet, nematode feeding group, morphology, DNA metabarcoding

## Abstract

Agricultural soil environments contain different types of nematodes in all trophic levels that aid in balancing the soil food web. Beneficial free-living nematodes (FLNs) consist of bacterivores, fungivores, predators, and omnivores that help in the mineralization of the soil and the top-down control of harmful plant-parasitic nematodes (PPNs). Annually, USD 125 billion in worldwide crop losses are caused by PPNs, making them a plant pathogen of great concern for growers. Farmers have started to implement the use of cover crops in agricultural systems for the protection and enrichment of soil but research on how different cover crops affect nematode populations is lacking and in demand. This study aims to determine the effects of legume and grass cover crops, Cowpea (*Vigna unguiculata*) and Pearl Millet (*Pennisetum glaucum*), as well as their mixture on the abundance and diversity of FLN and PPN populations. Soil samples were collected at the time of cover crop maturity and spinach harvest to analyze nematode communities using both morphological and DNA metabarcoding analysis. The results showed that the application of Cowpea and Pearl Millet as well as their mixture in a spinach agricultural system led to the control of PPNs and proliferation of FLN communities, with each cover crop treatment demonstrating different advantages for the various nematode feeding groups. Soil property analysis did not show a significant difference except for magnesium and total nitrogen levels, which were significantly correlated with nematode community composition. The overall findings of our study indicate that the choice of cover crop implementation by growers for spinach cultivation should be based on specific soil health conditions, which in turn promote soil fertility and a healthy nematode community.

## 1. Introduction

Nematodes are microscopic organisms under the large phylum *Nematoda* often found in microbial communities in soil and several other environments [1]. In soil environments, different types of nematodes are present, including free-living nematodes (FLNs) and plant-parasitic nematodes (PPNs). FLNs consist of several different feeding groups such as bacterivores, fungivores, omnivores, and predators [2]. Conversely, PPNs feed on plant roots to gain nutrients for survival. To pierce plant root tissues, PPNs use their stylet and may also secrete enzymes that aid in the degradation of the plant cell wall [2]. PPNs cause severe damage to agricultural crops resulting in costing USD 125 billion in crop losses worldwide [1]. Therefore, they are considered the world’s most damaging plant pathogen [3,4]. In Canada, PPNs may cause significant annual yield losses up to 15% [5].

A healthy agricultural soil consists of an environment with a good balance of beneficial FLNs as they contribute to nutrient cycling and the release of nitrogen in the soil [6,7]. Nematodes are also part of several trophic levels in the soil food web and play a crucial role in balancing the environment by feeding on various soil microorganisms and being a food source to other predators [6,8]. Changes in nematode population dynamics reflect changes in soil microbial communities making nematodes an important bioindicator of soil health and fertility [6,9]. To improve soil quality and crop yield in a sustainable manner, farmers have started implementing the use of cover crops during the off-season [10]. This allows the enrichment and protection of soil as well as suppression of weeds, disease, or pests [11]. Research has shown that different cover crops not only suppress PPN populations [12,13], but also increase FLN populations when incorporated into the soil [14,15]. This is because certain cover crops are non-hosts to the damaging PPNs [16]. *Brassica* cover crops have been found to suppress PPNs infection by hydrolysis of glucosinolate compounds found within the plant, a process termed biofumigation [17,18,19]. Studies have also shown that certain cover crops may serve as potential hosts to PPNs, allowing these populations to grow and cause further damage to the cash crop [20,21]. Growers should select cover crops that are poor or non-hosts to the PPNs of concern in their agricultural systems to promote a beneficial nematode community.

Although Cowpea (*Vigna unguiculata*) and Pearl Millet (*Pennisetum glaucum*) are commonly used cover crops in Ontario, Canada, their effect on FLN and PPN populations is largely unknown [22]. Furthermore, Pearl Millet may have a positive or negative influence on plant-parasitic nematodes and FLN [22,23]. In the current investigation, we aimed to gain a better understanding of the impact of cover crops on nematode communities, and how growers can mitigate crop yield losses by implementing the most beneficial cover crops to their agricultural systems. The main objectives of this study were to determine the effect of legume (Cowpea) and non-legume (Pearl Millet) cover crops and their mixture on the abundance, structure, and diversity of both FLN and PPN communities in a spinach cultivation system.

## 2. Results

**Figure 1:**
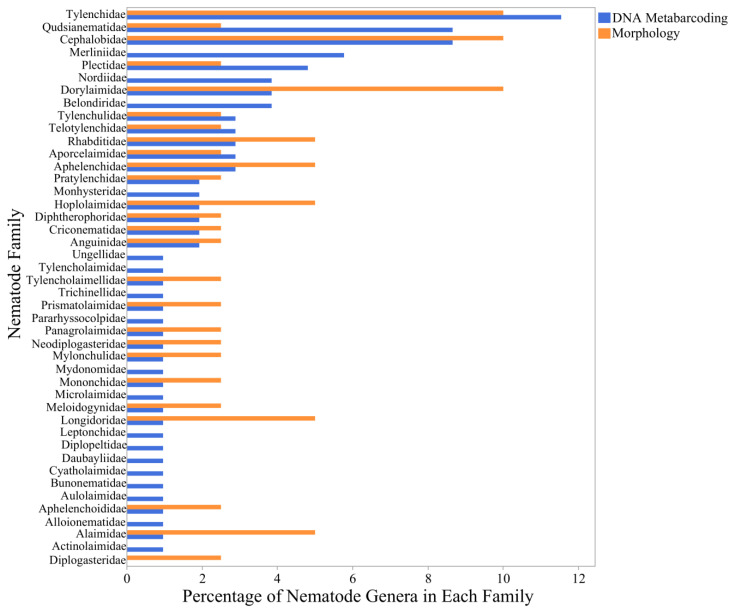
Percentage of nematode genera identified for each nematode family using morphological and DNA metabarcoding analyses.

Using the morphological and DNA metabarcoding analyses, 42 nematode families were identified in all soil samples (Figure 1). Of these 42 families, 41 were identified using the DNA metabarcoding method and 25 were identified using morphological analysis, with 24 families found by both methods. Most nematode families identified only using DNA metabarcoding were generally found to represent the rare taxa, with 1% of different nematode genera detected within the families of Tylencholaimidae, Tylencholaimellidae, Trichinellidae, Prismatolaimidae, Pararhyssocolpidae, Panagrolaimidae, Neodiplogasteridae, Mylonchulidae, Mydonomidae, Mononchidae, Monhysteridae, Microlaimidae, Meloidogynidae, Longidoridae, Leptonchidae, Daubayliidae, Cyatholaimidae, Bunonematidae, Aulolaimidae, Aphelenchoididae, Alloionematidae, Alaimidae, and Actinolaimidae (Figure 1).

The most diverse nematode families that were well-represented by both DNA metabarcoding and morphological analysis included Tylenchidae (12.3% of different nematode genera for DNA metabarcoding and 10.8% for morphology) and Cephalobidae (9.2% and 10.8%, respectively). Certain nematode families were over-represented in one identification method (Qudsianematidae, 9.2% for DNA metabarcoding vs. 2.7% for morphology; Dorylaimidae, 4.1% vs. 8.1%, respectively), while other families showed similar representation using both DNA metabarcoding and morphological analysis (Tylenchulidae, 2% vs. 2.7%; Pratylenchidae, 2% vs. 2.7%) (Figure 1).

**Figure 2:**
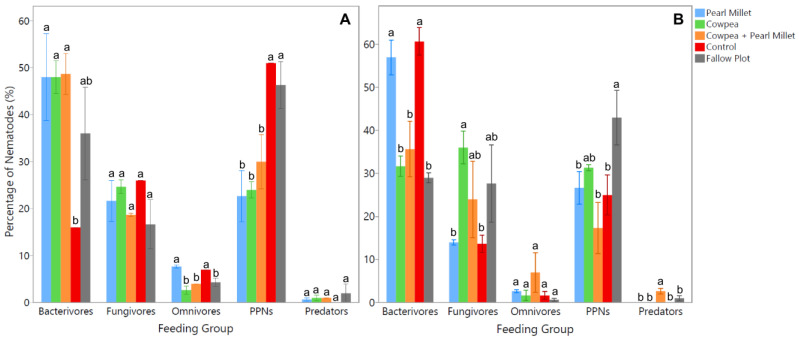
Average percentage of nematodes identified using morphological analysis in each feeding group during cover crop maturity (**A**), and spinach harvest time (**B**). Error bars are constructed using the standard error of means. Cover crop treatments within feeding groups not represented by the same letter are significantly different (ANOVA, *p* < 0.05; Student’s *t* test, *p* < 0.05).

Extracted nematodes from soil samples were examined under the microscope to identify species present based on morphological characteristics and classify them into respective feeding groups. Results from the time of cover crop maturity (Time 1) showed significant differences (*p* < 0.05) between cover crop treatments for the bacterivores, herbivores (PPNs), and omnivores (Figure 2A). The average number of bacterivores was found to be significantly higher (*p* < 0.05) in the Cowpea, Pearl Millet, and mixture of both cover crop treatments, having 100% more bacterivores than the Control. Conversely, the average number of PPNs was observed to be significantly higher (*p* < 0.05) in the Control and Fallow Plot, with 53% more PPNs than the mixture of Cowpea and Pearl Millet and 67% more PPNs than the Pearl Millet and Cowpea treatments (Figure 2A). In terms of omnivores, the average number was significantly higher (*p* < 0.05) in the Pearl Millet and Control treatments compared to all other experimental treatments, both having 67% more omnivores. No significant differences were observed between treatments for the fungivore and predator feeding groups at the time of cover crop maturity.

Results from the spinach harvest time (Time 2) showed significant differences (*p* < 0.05) between cover crop treatments for the bacterivores, herbivores (PPNs), and predators (Figure 2B). The average number of bacterivores was found to be significantly higher (*p* < 0.05) in the Control, with 53% more bacterivores than the mixture of Cowpea and Pearl Millet and 67% more than Cowpea and the Fallow Plot. Pearl Millet also had significantly more (*p* < 0.05) bacterivores than the mixture of covers with a difference of 49% and the same for Cowpea and Fallow Plot with a difference of 64%. The Fallow Plot had significantly higher (*p* < 0.05) PPNs than Pearl Millet and the Control by 44% and the mixture of Cowpea and Pearl Millet by 84% (Figure 2B). The average number of predators showed the opposite trend, as the mixture of cover crops had significantly more (*p* < 0.05) predators than Pearl Millet, Cowpea, the Control, and Fallow Plot by at least 100%. The average number of fungivores was significantly higher (*p* < 0.05) in the Cowpea treatment than Pearl Millet and the Control by at least 80%. No significant differences were observed between cover crop treatments for the omnivore feeding group at the spinach harvest time. Thus, the implementation of cover crops significantly influenced soil nematode communities with Cowpea, Pearl Millet, and their mixture largely increasing FLNs such as bacterivores, and the Control and Fallow Plot generally increasing PPNs.

**Figure 3:**
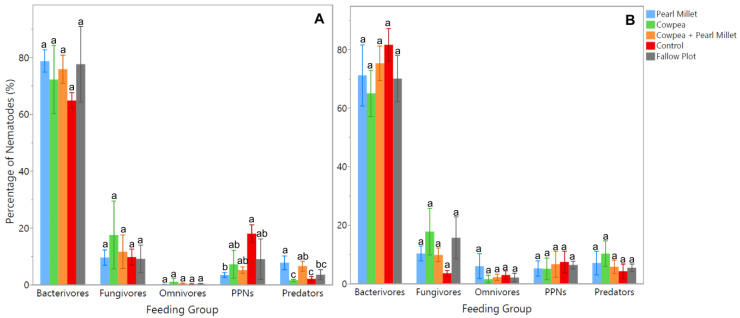
Average percentage of nematode OTUs (rarefied to 39,022 reads per sample) in each feeding group during cover crop maturity (**A**), and spinach harvest time (**B**). Error bars are constructed using the standard error of means. Cover crop treatments within feeding groups not represented by the same letter are significantly different (ANOVA, *p* < 0.05; Student’s *t* test, *p* < 0.05).

Soil samples were also analyzed using DNA metabarcoding analysis in order to have a broader understanding of nematode populations and soil health for each of the cover crop treatments. Results from the time of cover crop maturity (Time 1) exhibited significant differences (*p* < 0.05) between treatments for the herbivores (PPNs) and predators (Figure 3A). The average number of PPNs was noticed to be significantly higher (*p* < 0.05) in the Control treatment, with 120% more PPNs than Pearl Millet. On the other hand, the average number of predators was found to be significantly higher (*p* < 0.05) in Pearl Millet, with 107% more predators than the Fallow Plot and 133% more than Cowpea and the Control (Figure 3A). No significant differences were noted between treatments for the bacterivore, fungivore, and omnivore feeding groups at the time of cover crop maturity. The results from the time of spinach harvest (Time 2) showed no significant differences between cover crop treatments for any of the feeding groups (Figure 3B).

**Figure 4:**
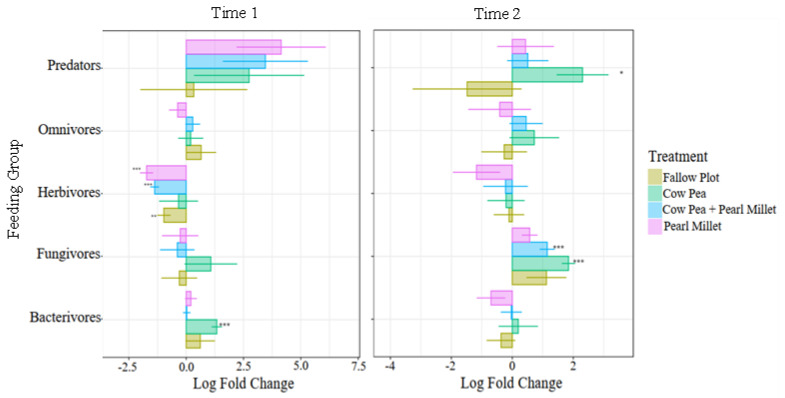
Differential abundance of feeding groups for each of the cover crop treatments compared to the Control treatment (intercept) with mean log fold change at cover crop maturity (Time 1) and spinach harvest (Time 2). Output is derived from Analysis of Compositions of Microbiomes with Bias Correction (ANCOM −BC). Standard error bars are shown. Cover crops within feeding groups significantly different from the Control (*q* < 0.05, Holm-adjusted *p*-value) are indicated (* = 0.05, ** = 0.01, *** = 0.001).

In the study, the log fold differences of feeding groups in the different cover crop treatments were compared to the Control using ANCOM-BC (Figure 4). With mature cover crops present (Time 1), we found that the relative abundance of herbivores (PPNs) decreased significantly in the Pearl Millet (−1.7 log_e_), mixture of Cowpea and Pearl Millet (−1.4 log_e_), and the Fallow (−1.0 log_e_) treatments. Bacterivores were only found to increase in Cowpea (1.3 log_e_) with mature cover crops (Time 1) and were not differentially abundant by spinach harvest (Time 2). At the spinach harvest time, there was a significant increase in predators for Cowpea (2.3 log_e_) and a significant increase in fungivores for Cowpea (1.8 log_e_) and the Cowpea and Pearl Millet mixture (1.1 log_e_) (Figure 4).

In the present work, ecological indices were calculated for all sets of soil samples, which included richness, representing the number of species present; the Shannon–Wiener Index (SWI) value, a measure of species diversity; and the Bonger’s Maturity Index (MI), a measure of environmental disturbance based on nematode species composition. Ecological indices values for Time 1 described the soil environment for treatments after cover crops were seeded and given the appropriate amount of time to mature (Table 1).

The results at cover crop maturity (Time 1) revealed that there were no significant differences between richness, or SWI values for all cover crop treatments (Table 1). The MI value of the Control treatment (2.14) was significantly higher (*p* < 0.05) by 22% in comparison with the mixture of Cowpea and Pearl Millet as well as the Cowpea treatments (1.71). These results indicated that the soil in the Control treatment was less disturbed. However, there was no significant difference in MI using metabarcoding. There were no significant differences between the richness, SWI, or MI values for all cover crop treatments at the time of spinach harvest (Time 2).

**Figure 5:**
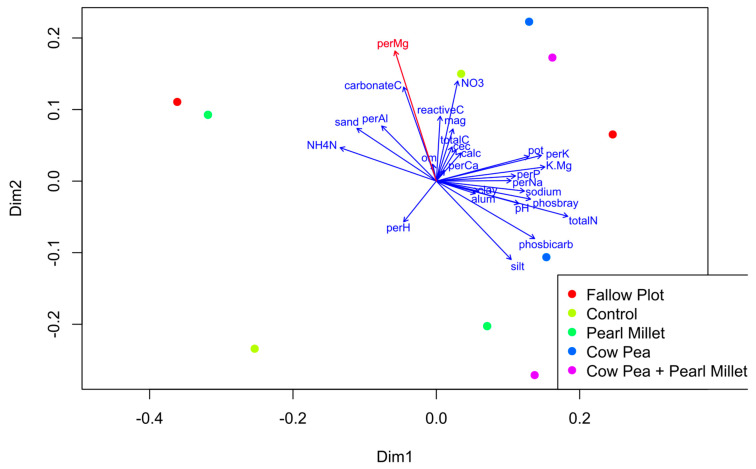
Plotting soil environmental variables onto Principal Coordinates Analysis (PCoA) ordination from DNA metabarcoding. Colours represent different cover crop treatments and times (as Figure 2, Figure 3 and Figure 4). Percent magnesium (red arrow) was found to have the highest correlation coefficient (*p* = 0.04). Significance was tested by 999 permutations. PC1 (Dim1) and PC2 (Dim2) represent the first two principal components.

Analysis of soil properties was also performed on all samples to obtain a broader understanding of how they are correlated with the nematode community composition in cover crop treatments. The envfit function was used to fit soil properties onto the Principal Coordinates Analysis (PCoA) ordination from DNA metabarcoding data. The results from all soil properties showed that the percentage of magnesium (Mg) was significantly correlated to the ordination axes (r^2^ = 0.59, *p* = 0.04) and was generally higher in samples taken during cover crop maturity (Time 1) (Figure 5). Total nitrogen (totalN) showed the second highest correlation coefficient with the ordination axes (r^2^ = 0.58, *p* = 0.07) being significantly correlated to the community composition by a Mantel test (*p* = 0.02) at cover crop maturity.

## 3. Discussion

The present investigation identified 42 nematode families across all studied soil samples using both morphological and DNA metabarcoding analyses. The DNA metabarcoding approach detected a broader range of nematode families, identifying 41 families compared to the 25 found through morphological analysis, with 24 families common to both methods. This discrepancy highlights the greater sensitivity of the DNA metabarcoding method, particularly in detecting the rare taxa. Tylencholaimidae, Tylencholaimellidae, and a few other families were detected only by DNA metabarcoding. This may be due to their lower abundance, making them less visible to traditional morphological approaches. The rare taxa, while not abundant, may play crucial roles in soil ecosystems, and their detection underscores the importance of integrating molecular techniques in nematode community studies [24,25].

Among the families well-represented by both methods, Tylenchidae and Cephalobidae stood out, comprising significant portions of the nematode communities (12.3% and 9.2% for DNA metabarcoding, and 10.8% each for morphology). The consistency in detecting these families across methods validates the reliability of the morphological approach for more common and morphologically distinctive nematodes. However, discrepancies such as the over-representation of Qudsianematidae in DNA metabarcoding (9.2% vs. 2.7% in morphology) and the higher detection of Dorylaimidae through morphological analysis (8.1% vs. 4.1% in DNA metabarcoding) suggest that each method has biases, potentially linked to the differing sensitivities to DNA extraction efficiency and morphological identification challenges [26,27].

The temporal dynamics of nematode communities were also explored by comparing cover crop treatments at two key time points: cover crop maturity and spinach harvest. Morphological analysis at cover crop maturity revealed significant variations in the abundance of different feeding groups. Bacterivores were notably more abundant in Cowpea, Pearl Millet, and their mixture, showing a 100% increase compared to the Control. This suggests that these cover crops may enhance bacterial populations, which in turn support larger bacterivore communities, by providing organic matters. Furthermore, the higher abundance of bacterivores associated with cover crops indicates an accelerated nutrient cycling and a greater mineral nitrogen availability, which can enhance soil fertility and support the growth of the subsequent cash crop [6]. Conversely, PPNs were significantly more abundant in the Control and Fallow plots, with 53% more PPNs than in the mixture of Cowpea and Pearl Millet, and 67% more than in the individual Cowpea and Pearl Millet treatments. This indicates that cover crops created enriched environments that favor the proliferation of suppressive organisms such as fungi, bacteria, and predatory nematodes that, in turn, can prey on and suppress PPN populations [28,29,30]. Our results are consistent with past studies which found that legume and non-legume cover crop mixes often provide better PPN suppression that compared to single species [30,31,32]. However, the non-legume cover crops included in most studies consist of *Brassica* spp., as they contain glucosinolate compounds that suppress PPN infection [33,34]. Omnivores, on the other hand, were more prevalent in the Pearl Millet and Control treatments, which may reflect the broader trophic flexibility of this group in these particular soil environments.

The nematode community composition shifts by spinach harvest time. Bacterivores were now the most abundant in the Control, suggesting a decline in bacterial activity in cover-cropped soils as the season progressed. Interestingly, Pearl Millet still supported more bacterivores than the cover crop mixture, possibly due to differences in root exudate composition or microbial community structure between these treatments. PPNs were most numerous in the Fallow Plot, which lacked the protective influence of cover crops, leading to higher pest pressure. Predators, crucial for natural nematode pest control, were significantly more abundant in the cover crop mixture; highlighting the potential of using diverse cover cropping to enhance predatory nematode populations and contribute to biological control. Research has shown that combining more than one cover crop can lead to increased multi-functionality of covers and improved outcomes compared to implementing a monoculture in an agricultural system [32,35]. Legume and grass cover crop mixtures are found as the most effective combinations for producing high-quality residues in agricultural soil due to the complementarity of both crop types [29].

DNA metabarcoding corroborated these findings with additional insights. PPNs were significantly more abundant at cover crop maturity in the Control treatment, with a 120% increase compared to Pearl Millet. This emphasizes the suppressive effect of Pearl Millet on nematode pests. Predators, on the other hand, were 107% more abundant in Pearl Millet compared to the Fallow Plot and 133% more than in Cowpea and the Control, reinforcing the idea that certain cover crops can enhance beneficial nematode populations. The higher abundance of predatory nematodes in the Pearl Millet treatment could also explain the lower abundance of PPNs in this same treatment, as predatory nematodes provide top-down control of these damaging populations [36]. No significant differences were observed by spinach harvest among treatments for any feeding groups, suggesting a convergence in nematode community composition as the cropping cycle progressed.

The differential abundance analysis provided further depth by showing that the relative abundance of PPNs decreased significantly in Pearl Millet, the Cowpea and Pearl Millet mixture, and Fallow treatments, confirming the suppressive effect of these cover crops. On the contrary, bacterivores increased significantly in Cowpea at cover crop maturity, reflecting the promotion of bacterial populations under this cover crop [32]. Predators increased significantly by spinach harvest in Cowpea, and fungivores were more abundant in both Cowpea and the cover crop mixture, highlighting the dynamic shifts in soil food webs influenced by different cover cropping strategies. The mixture of cover crops also revealed significantly higher fungivores than the Control at this time-point. Fungivores are an important nematode feeding group as they feed on plant-pathogenic fungi, reducing the risk of crop damage by these microorganisms [37].

Ecological indices, including richness, SWI, and the MI, were calculated to assess broader soil ecosystem health. At cover crop maturity, there were no significant differences in richness or SWI among treatments, demonstrating that overall species diversity remained stable across different cover crop treatments. However, the MI value was significantly higher in the Control treatment in the morphology data, suggesting that the soil in this treatment was less disturbed, potentially due to the absence of cover crops, which can disrupt nematode communities through changes in root structure and soil microenvironment [38,39,40]. Spinach harvest was the time point by which no significant differences were observed in any of the ecological indices, suggesting that the initial effects of cover cropping on nematode communities had diminished over time; this may be attributed to the uniformity imposed by subsequent agricultural practices. Soil property analysis revealed that magnesium levels were significantly correlated with nematode community composition, particularly at cover crop maturity. This finding, along with the significant correlation of total nitrogen with community composition, underscores the complex interactions between soil chemistry and biological communities, influenced by cover crop selection and management practices.

## 4. Materials and Methods

### 4.1. Experimental Design and Field Maintenance

The experimental field for this study was located in St. Thomas, ON, Canada in the Lake Erie Basin. At this site, common cropping practices and pest management measures were used for the maintenance of the crops without additional fertilizer applications. The experimental field (55 m by 10 m) comprised five treatments arranged in a randomized complete block design with three replicating blocks. The cover crop treatments included Cowpea (*Vigna unguiculata*), Pearl Millet (*Pennisetum glaucum*), mixture of Cowpea and Pearl Millet, Control (spinach cultivation only), and Fallow Plot (no spinach or cover crop). Cowpea and Pearl Millet cover crops were planted prior to the plantation of the main crop, spinach. After cover crops were seeded, they were given about four weeks to grow and mature before being tilled to incorporate the organic matter into the soil. Spinach was then planted and given the time to mature before being harvested.

### 4.2. Collecting Soil Samples and Extracting Nematodes

Soil samples were collected from each sub-plot at two different periods for a total of 15 soil samples per sampling or 30 soil samples in total. The first set of samples was collected after seeding cover crops and allowing them to mature in August 2021 (Time 1). Samples taken at this time were used to determine how the different cover crops affect overall nematode populations. The second set of samples was taken after cover crops were tilled into the soil and the spinach was mature and ready to be harvested in September 2021 (Time 2). This design allowed a better understanding of how the nematode population changed after plantation of the main crop. During soil sample collection, 30 cm soil probes were used to take 10 random cores of soil in each sub-plot to obtain a good representation of the nematode population for the entire treatment. These 10 sub-samples were then put into a single sampling bag to create one composite sample for the sub-plot. Soil probes were cleaned between the sampling of each sub-plot to avoid cross-contamination. Once soil samples were collected, they were placed directly in a cooler with ice to ensure that nematodes do not overheat and die during transportation from the field to the laboratory. All soil samples were stored at 4 °C and nematodes were extracted from soil samples using sugar centrifuge techniques [30], and preserved in 5% formalin until morphological analysis.

### 4.3. Morphological Analysis

One hundred hand-picked nematodes were obtained from each soil sample and identified with Leica stereo microscope (MC205 C, Concord, ON, Canada). Nematodes were identified to the family or genus level using the diagnostic keys of the University of Nebraska nematode identification website (https://nematode.unl.edu/nemaID.htm, accessed on 5 February 2024). Nematodes were grouped into bacterivores, fungivores, plant-parasitic nematodes, omnivores, and predators based on their mouthparts.

### 4.4. DNA Metabarcoding Analysis

Nematode DNA was extracted from nematodes using the QIAGEN DNeasy^®^ Blood and Tissue Kit (Catalog #69506, Qiagen, Mississauga, ON, Canada), following the manufacturer’s instructions. Extracted DNA was sent to Genome Quebec for DNA metabarcoding along with the nematode specific primers NF1-18Sr2b or NEM [41], with paired-end (PE300) sequencing performed using an Illumina MiSeq platform (Génome Québec, Montréal, QC, Canada)The raw reads were uploaded onto the high-performance computing (HPC) servers for data analysis using an in-house bioinformatics pipeline. The process consisted of three major steps: (1) preprocessing using the fastp tool [42] with the default setting to trim primer and adaptor sequences, filter out low quality and long soft clipping reads, and eliminate any chimeric sequences; (2) aligning the processed sequence reads for individual samples to the nematode 18S rRNA reference sequences using bwa (mem) [43] with the default settings, sorted and indexed (without PCR duplicate removal) using samtools [44]; and (3) processing the mapping output in binary sequence alignment mapping format (BAM) using an in-house PERL script to select qualified reads by requiring a minimal read length of 100 bp with a minimal alignment score of 100, a maximal number of mismatches of five, and a maximal length of soft clipping at 10 bp. The output of the pipeline is the number of mapped reads per species in each sample. A sequence read from a sample was used only once based on the best alignment, and a taxonomic group requires a minimum of two reads to be reported. The SILVA sequence database (v138) [45] was used for retrieving the nematode 18S rRNA reference sequences (converted to DNA sequences) for read mapping and for taxonomic associations of nematode species present in all samples. Prior to diversity analysis, the samples were rarefied to an even number of reads per sample (39,022 reads). Alpha rarefaction was computed to help confirm this cut-off was appropriate.

### 4.5. Statistical Analysis

Data analyses were performed in R software version 4.3.1 [46] and JMP^®^ v.17 (SAS Institute Inc., Cary, NC, USA). The vegan package was utilized for rarefying DNA metabarcoding results to reduce sequencing bias in the OTU outputs before data normalization and analysis [47]. The significance of the effects of legume and non-legume cover crops, as well as their mixtures, at two different time points on the abundance and diversity of FLN and PPN communities was assessed by analysis of variance (ANOVA). The mixed model with time and treatment as variables and blocking as random effect was used to analyze the data. Normality was confirmed by visual inspection of the model’s residuals and using the Shapiro–Wilk test. In the event of non-normality, we applied either a square-root or log transformation to the dataset before proceeding with the analysis. A *p*-value of 0.05 was used as a threshold of acceptance of the significance of effects. The significance of the differences between treatment means was assessed using the LSMeans Student’s *t* tests.

Ecological indices were calculated to further analyze and interpret results. Bonger’s Maturity Index (MI), which is a measure of environmental disturbance based on the composition of non-plant feeding nematode species present in the soil, was calculated [14]. A low MI score indicates a more disturbed soil environment, whereas a higher MI score indicates a more stable soil environment [14]. Richness was calculated for each cover crop treatment in both morphological and DNA metabarcoding data. The Shannon–Wiener Diversity Index (SWI) was also calculated for both morphological and DNA metabarcoding results to interpret nematode diversity in the different soil samples [36]. The significance of the differences between treatment means for ecological indices was also evaluated using the LSMeans Student’s *t* tests option.

Differential abundance of feeding groups for DNA metabarcoding data was performed using Analysis of Compositions of Microbiomes with Bias Correction (ANCOM-BC) in the R package ‘ancombc’ [48]. The bias-corrected absolute log (log_e_) fold changes were plotted as compared to the Control treatment.

Nematode community composition (ASV data were pooled from the three blocks) was visualized using Principle Coordinates Analysis (PCoA) of the Canberra dissimilarity matrix, implemented in ‘vegan’ [49]. Environmental variables were then projected onto the ordination using envfit (vegan), which calculates the multiple regression of environmental variables with ordination axes, and the significance is tested by permutation.

## 5. Conclusions

Our research reveals the advantages and limitations of morphological and DNA metabarcoding approaches when used to study nematode communities in soil ecosystems. Combining both methods results in a more comprehensive understanding of the nematode communities. Cover crops enhance soil health, proliferating FLNs, and managing PPNs, especially when mixtures of legume and non-legume species are used. Cover crop mixtures offer broader ecosystem benefits compared to monocultures like Pearl Millet, which may have limited benefits in terms of overall ecosystem health and nutrient cycling improvement due to their narrower focus on specific pests. The changes in nematode populations from cover crop growth to spinach harvest show how time plays a crucial role in maintaining soil health through sustainable cover crop strategies that balance crop production and soil quality.

## Figures and Tables

**Table 1:** Ecological indices for samples analyzed with morphological and DNA metabarcoding analysis at the time of cover crop maturity (Time 1) and spinach harvest (Time 2). The standard error values are included for every calculated average. Values not represented by the same letter are significantly different (ANOVA, *p* < 0.05; Student’s *t* test, *p* < 0.05).

	Cover Crop Maturity (Time 1)					Spinach Harvest (Time 2)				
	Pearl Millet	Cowpea	Cowpea + Pearl Millet	Control	Fallow Plot	Pearl Millet	Cowpea	Cowpea + Pearl Millet	Control	Fallow Plot
Richness (Morphology)	17 ± 1.15 (a)	18 ± 2.52 (a)	20 ± 4.04 (a)	12 ± 0 (a)	17 ± 5.69 (a)	10 ± 1.53 (a)	11 ± 1.73 (a)	15 ± 4.58 (a)	15 ± 3 (a)	15 ± 2.08 (a)
Richness (DNA Metabarcoding)	106 ± 5.72 (a)	106 ± 12.36 (a)	116 ± 21.75 (a)	88 ± 23.42 (a)	81 ± 15.58 (a)	123 ± 24.85 (a)	102 ± 5.35 (a)	125 ± 19.82 (a)	110 ± 23.23 (a)	114 ± 7.48 (a)
Shannon–Wiener Diversity Index (SWI) (Morphology)	2.37 ± 0.12 (a)	2.39 ± 0.068 (a)	2.48 ± 0.19 (a)	2.12 ± 0 (a)	2.33 ± 0.21 (a)	1.96 ± 0.14 (a)	1.98 ± 0.17 (a)	2.29 ± 0.61 (a)	2.07 ± 0.061 (a)	2.04 ± 0.11 (a)
Shannon–Wiener Diversity Index (SWI) (DNA Metabarcoding)	2.59 ± 0.019 (a)	2.57 ± 0.26 (a)	2.87 ± 0.16 (a)	2.57 ± 0.58 (a)	2.08 ± 0.72 (a)	2.76 ± 0.78 (a)	2.75 ± 0.51 (a)	2.49 ± 0.50 (a)	2.53 ± 0.21 (a)	2.53 ± 0.44 (a)
Bonger’s Maturity Index (MI) (Morphology)	1.82 ± 0.13 (bc)	1.71 ± 0.12 (c)	1.71 ± 0.088 (c)	2.14 ± 0 (a)	1.99 ± 0.19 (ab)	1.77 ± 0.16 (a)	1.71 ± 0.29 (a)	1.82 ± 0.41 (a)	1.41 ± 0.015 (a)	1.81 ± 0.066 (a)
Bonger’s Maturity Index (MI) (Metabarcoding)	1.79 + 0.50 (a)	1.27 + 0.33 (a)	1.19 + 0.04 (a)	1.51 ± 0.14 (a)	1.51 + 0.31 (a)	1.27 + 0.14 (a)	1.53 + 0.31 (a)	1.51 + 0.14 (a)	1.48 + 0.29 (a)	1.41 + 0.24 (a)

## Data Availability

The data presented in this study are available on request from the corresponding author. The data are not publicly available due to government restrictions.

## References

[1] Mesa-Valle C.M., Garrido-Cardenas J.A., Cebrian-Carmona J., Talavera M., Manzano-Agugliaro F. (2020). Global research on plant nematodes. Agronomy.

[2] Iqbal S., Jones M.G.K., Thomas B., Murray B.G., Murphy D.J. (2017). Nematodes. Encyclopedia of Applied Plant Sciences.

[3] Abd-Elgawad M., Askary T. (2015). Impact of Phytonematodes on agriculture Economy.

[4] Sikora R.A., Molendijk L.P.G., Desaeger J. (2021). Integrated nematode management and crop health: Future challenges and opportunities. Integrated Nematode Management: State-of-the-Art and Visions for the Future.

[5] Potter J.W., McKeown A.W. (2003). Nematode biodiversity in Canadian agricultural soils. Can. J. Soil Sci..

[6] Melakeberhan H., Bonito G., Kravchenko A.N. (2021). Application of nematode community analyses-based models towards identifying sustainable soil health management outcomes: A review of the concepts. Soil Syst..

[7] van den Hoogen J., Routh D., Ferris H., Traunspurger W., Wardle D.A., de Goede R.G.M., Adams B.J., Ahmad W., Andriuzzi S.W., Bardgett R.D. (2019). Soil nematode abundance and functional group composition at a global scale. Nature.

[8] Ferris H., Venette R.C., Scow K.M. (2004). Soil management to enhance bacterivore and fungivore nematode populations and their nitrogen mineralisation function. Appl. Soil Ecol..

[9] Khanum T., Mahmood N. (2021). Bacterial Feeding Nematodes Use for Nitrogen Mineralization and Plant Production. Acta Sci. Pharm. Sci..

[10] Holland J., Brown J.L., MacKenzie K., Neilson R., Piras S., McKenzie B.M. (2021). Over winter cover crops provide yield benefits for spring barley and maintain soil health in northern Europe. Eur. J. Agron..

[11] Ingels C., Horn M., Bugg R., Miller P. (1994). Selecting the right cover crop gives multiple benefits. Calif. Agric..

[12] Fageria N.K., Baligar V.C., Bailey B.A. (2007). Role of cover crops in improving soil and row crop productivity. Commun. Soil Sci. Plant Anal..

[13] Thapa R., Mirsky S.B., Tully K.L. (2018). Cover crops reduce nitrate leaching in agroecosystems: A global meta-analysis. J. Environ. Qual..

[14] Bongers T., Bongers M. (1998). Functional diversity of nematodes. Appl. Soil Ecol..

[15] DuPont S.T., Ferris H., Van Horn M. (2009). Effects of cover crop quality and quantity on nematode-based soil food webs and nutrient cycling. Appl. Soil Ecol..

[16] Bélair G., Forge T., Mimee B., Tenuta M., Yu Q., Subbotin S.A., Chitambar J.J. (2018). Current State of Plant Parasitic Nematodes in Canada. Plant Parasitic Nematodes in Sustainable Agriculture of North America: Vol.1—Canada, Mexico and Western USA.

[17] Agerbirk N., Olsen C.E. (2012). Glucosinolate structures in evolution. Phytochemistry.

[18] Gimsing A.L., Kirkegaard J.A. (2009). Glucosinolates and biofumigation: Fate of glucosinolates and their hydrolysis products in soil. Phytochem Rev..

[19] Van Dam N.M., Tytgat T.O.G., Kirkegaard J.A. (2009). Root and shoot glucosinolates: A comparison of their diversity, function and interactions in natural and managed ecosystems. Phytochem. Rev..

[20] Neupane K., Yan G. (2023). Host Suitability of Cover Crops to the Root-Lesion Nematode Pratylenchus penetrans Associated with Potato. Plant Dis..

[21] Hashemi K., Karegar A. (2018). Host suitability of common agricultural crops to Scutylenchus rugosus (Siddiqi, 1963) Siddiqi, 1979 in Iran, with a focus on wheat and maize. Nematology.

[22] Bélair G., Dauphinais N., Fournier Y., Dangi O.P., Ciotola M. (2006). Effect of 3-year rotation sequences and pearl millet on population densities of Pratylenchus penetrans and subsequent potato yield. Can. J. Plant Pathol..

[23] Diakhaté S., Villenave C., Diallo N.H., Ba A.O., Djigal D., Masse D., Sembène P.M., Chapuis-Lardy L. (2013). The influence of a shrub-based intercropping system on the soil nematofauna when growing millet in Senegal. Eur. J. Soil Biol..

[24] Griffiths B.S., de Groot G.A., Laros I., Stone D., Geisen S. (2018). The need for standardisation: Exemplified by a description of the diversity, community structure and ecological indices of soil nematodes. Ecol. Indic..

[25] Kawanobe M., Toyota K., Ritz K. (2021). Development and application of a DNA metabarcoding method for comprehensive analysis of soil nematode communities. Appl. Soil Ecol..

[26] Floyd R., Abebe E., Papert A., Blaxter M. (2002). Molecular barcodes for soil nematode identification. Mol. Ecol..

[27] Powers T., Harris T., Higgins R., Mullin P., Sutton L., Powers K. (2011). MOTUs, morphology, and biodiversity estimation: A case study using nematodes of the suborder criconematina and a conserved 18S DNA barcode. J. Nematol..

[28] Dabney S.M., Delgado J.A., Reeves D.W. (2001). Using winter cover crops to improve soil and water quality. Commun. Soil Sci. Plant Anal..

[29] de Araujo F.G., Teixeira J.C.S., de Souza C.J., Arieira R.D.C. (2023). Cover crops and biocontrol agents in the management of nematodes in soybean crop. Rev. Caatinga.

[30] Akanwari J., Islam M.R., Sultana T. (2024). The Impact of Winter Cover Crops on Soil Nematode Communities and Food Web Stability in Corn and Soybean Cultivation. Microorganisms.

[31] Boudreau M.A. (2013). Diseases in intercropping systems. Annu. Rev. Phytopathol..

[32] Chapagain T., Lee E.A., Raizada M.N. (2020). The Potential of multi-species mixtures to diversify cover crop benefits. Sustainability.

[33] Potter M.J., Davies K., Rathjen A.J. (1998). Suppressive Impact of Glucosinolates in Brassica Vegetative Tissues on Root Lesion Nematode *Pratylenchus neglectus*. J. Chem. Ecol..

[34] Snapp S.S., Swinton S.M., Labarta R., Mutch D., Black J.R., Leep R., Nyiraneza J., O’Neil K. (2005). Evaluating cover crops for benefits, costs and performance within cropping system niches. Agron. J..

[35] Finney D.M., Murrell E.G., White C.M., Baraibar B., Barbercheck M.E., Bradley B.A., Cornelisse S., Hunter M.C., Kaye J.P., Mortensen D.A. (2017). Ecosystem services and disservices are bundled in simple and diverse cover cropping systems. Agric. Environ. Lett..

[36] Li J., Peng P., Zhao J. (2020). Assessment of soil nematode diversity based on different taxonomic levels and functional groups. Soil Ecol. Lett..

[37] Nicholas W.L. (1975). The Biology of Free-Living Nematodes.

[38] Cakmak I. (2013). Magnesium in crop production, food quality and human health. Plant Soil.

[39] Huber D.M., Jones J.B. (2013). The role of magnesium in plant disease. Plant Soil.

[40] Senbayram M., Gransee A., Wahle V., Thiel H. (2015). Role of magnesium fertilisers in agriculture: Plant–soil continuum. Crop Pasture Sci..

[41] Porazinska D.L., Giblin-Davis R.M., Faller L., Farmerie W., Kanzaki N., Morris K., Powers T.O., Tucker A.E., Sung W., Thomas W.K. (2009). Evaluating high-throughput sequencing as a method for metagenomic analysis of nematode diversity. Mol. Ecol. Resour..

[42] Chen S., Zhou Y., Chen Y., Gu J. (2018). Fastp: An ultra-fast all-in-one FASTQ preprocessor. Bioinformatics.

[43] Li H. (2013). Aligning sequence reads, clone sequences and assembly contigs with BWA-MEM. arXiv.

[44] Danecek P., Bonfield J.K., Liddle J., Marshall J., Ohan V., Pollard M.O., Whitwham A., Keane T., McCarthy S.A., Davies R.M. (2021). Twelve years of SAMtools and BCFtools. GigaScience.

[45] Quast C., Pruesse E., Yilmaz P., Gerken J., Schweer T., Yarza P., Peplies J., Glöckner F.O. (2012). The SILVA ribosomal RNA gene database project: Improved data processing and web-based tools. Nucleic Acids Res..

[46] R Core Team (2024). R: A Language and Environment for Statistical Computing.

[47] Dixon P. (2003). VEGAN, a package of R functions for community ecology. J. Veg. Sci..

[48] Lin H., Peddada S.D. (2020). Analysis of compositions of microbiomes with bias correction. Nat. Commun..

[49] Oksanen J., Simpson G., Blanchet F.G., Kindt R., Legendre P., Minchin P., Hara R., Solymos P., Stevens M.H.H., Szoecs E. (2024). Vegan Community Ecology Package Version 2.6-8. https://cran.r-project.org/web/packages/vegan/vegan.pdf.

